# Effects of Ambient pH on the Growth and Development, Pathogenicity, and Diacetoxyscirpenol Accumulation of Muskmelon Fruit Caused by *Fusarium sulphureum*

**DOI:** 10.3390/jof10110765

**Published:** 2024-11-03

**Authors:** Qili Liu, Lan Yang, Huali Xue, Yang Bi, Qianqian Zhang, Yuanyuan Zong, Xiao Li

**Affiliations:** 1 College of Food Science and Engineering, Gansu Agricultural University, Lanzhou 730070, China; 2 College of Science, Gansu Agricultural University, Lanzhou 730070, China

**Keywords:** *Fusarium sulphureum*, muskmelon fruit, pathogenicity, mycotoxin, cell wall-degrading enzymes

## Abstract

Ambient pH, an important environmental factor, affects the growth, pathogenicity, and mycotoxin production of pathogenic fungus. *Fusarium sulphureum* is one of the predominant causal agents causing fusarium rot of muskmelon. In this study, we investigated the effects of ambient pH on fusarium rot development and diacetoxyscirpenol (DAS) accumulation in muskmelon infected with *F. sulphureum*, then analyzed the possible mechanisms in vitro and in vivo. The results suggested that ambient pH 6 was more conducive to the growth, pathogenicity, and mycotoxin production of *F. sulphureum* in vitro. Ambient pH 6 was also more favorable for secretion of cell wall-degrading enzymes for the pathogen to degrade the cell wall of the host plant and up-regulated the relative expression of genes involved in DAS biosynthesis, thus aggravating fruit disease and DAS accumulation. However, when the pH of the inoculated spore suspension was too acidic or too alkaline, the opposite results were observed.

## 1. Introduction

*Fusarium sulphureum* is an important postharvest pathogenic fungus and can cause postharvest diseases of muskmelon and potato tuber [1,2]. Postharvest diseases caused by *F. sulphureum* not only deteriorate the postharvest quality of fruit and vegetables but also lead to diacetoxyscirpenol (DAS) contamination in fruit and vegetables. DAS is a highly toxic type A trichothecene that can contaminate food and animal feed, thus causing serious harm to human and animal health [3]. *F. sulphureum* invades mainly through wounds or natural openings, resulting in the colonization and spread of pathogenic fungi in plants, ultimately leading to the occurrence of disease. When the pathogens infect host plants, ambient pH plays an important role in the colonization and expansion of pathogens.

pH is one of the main environmental factors affecting the pathogenicity of fungi to some certain extent, and can affect the growth and development of pathogenic fungi [4]. Spore germination and sporulation are important indexes to measure the reproductive ability and spore survival ability of filamentous fungi. The central developmental pathway composed of three key developmental activators, *BrlA*, *AbaA*, and *WetA*, regulates asexual development and sporulation through the transduction of developmental signals. *BrlA* is a zinc-finger domain-containing transcription factor, and is also the hub of the central pathway and upstream regulatory factor signaling. *BrlA* is independent and necessary in developmental regulation. Activation of *BrlA* is an important marker of the initiation of asexual development and occurs in the early stage of conidiophore development [5]. The important role of *BrlA* after activation is to immediately activate *AbaA*, which is expressed in the middle stage of conidiophore development. *WetA* is an important regulator of spore maturation. In the late stage of conidiophore development, activated *AbaA* activates the expression of *WetA* [6]. The downstream central pathway of *VosA* exists in the nucleus of mature spores and is also an important factor regulating spore maturation, which is mainly responsible for the accumulation of trehalose in spores [7]. At the end of sporulation, the expression of *BrlA* is inhibited by their feedback, thus completing the whole asexual development process. Zhang et al. [8] found that different ambient pH had a significant effect on the mycelial growth and sporulation of *Ustilaginoidea virens*. The mycelium grew better at pH 4.5–7.0, while the mycelial growth was inhibited at pH ≤ 4.0 and pH ≥ 7.5. When the ambient pH was 5.0, sporulation was at its highest. Similar results were found by Jimdjio et al. [9], who suggested that colony growth of *Penicillium expansum* was significantly inhibited in too acidic (2.5) or too basic (8.5) an environment.

In addition to influencing the growth and development of pathogenic fungi, ambient pH also affects the secretion and expression of pathogenic factors. Pathogenic fungi interfere with the normal physiological metabolism of host cells and destroy cells by secreting metabolites that are harmful to the host, such as cell wall-degrading enzymes (CWDEs), mycotoxins, and pathogenic hormones, which play crucial roles in pathogenicity [10]. CWDEs secreted by pathogenic fungi are enzymes that can degrade the host cell wall when infecting the host plant. CWDEs are considered to be the most important pathogenic factors in the pathogenesis of most postharvest diseases [11,12]. CWDEs are mainly composed of keratinase, pectinase, and cellulase. Pectin is an important CWDE, and is composed of polygalacturonase (PG), pectin methylase (PME), pectin methyl polygalacturonase (PMG), pectin lyase (PL), polygalacturonic acid trans-eliminase (PGTE), and pectin methyl-trans-eliminase (PMTE). PME first catalyzes the demethylation of the main chain of pectin in the cell wall and the middle layer of the fruit and produces a free carboxyl group while releasing methanol. The release of methanol leads to a decrease in the pH value of the cell wall, which is more conducive to the role of PG. PG can hydrolyze the α-1,4 glycosidic bond in the polygalacturonic acid in the cell wall and the middle glue layer, resulting in the destruction of the middle glue layer and the loosening of the cell wall structure, resulting in softening and decay of the fruit tissue. PMG is a hydrolase that specifically hydrolyzes the glycosidic bond of the substrate. It has a high selectivity for the degree of esterification of the substrate and can hydrolyze the α-1,4 glycosidic bond of highly esterified pectin esters. PL can catalyze the degradation of galacturonic acid and produce unsaturated oligosaccharides with 4-deoxy-α-D-galactose-4-enoic acid groups at its non-reducing end through β-elimination [13,14]. PGTE and PMTE are two important lyases and use pectin acid and pectin as substrates, respectively [15]. Cellulase is mainly composed of cellulase (Cx) and β-glucosidase (β-Glu). Cx is an important cell wall enzyme in the process of fruit softening, and can degrade cellulose and soften fruit. The increase in Cx activity is one of the main reasons for fruit softening. β-Glu mainly hydrolyzes β-D-glycosidic bonds at non-reducing ends to produce β-D-glucose [14]. Studies have shown that ambient pH affects the pathogenicity of fungi to fruit by regulating the secretion of CWDEs such as PG, PME, PMG, and PL [16,17]. In addition, ambient pH also plays a crucial role in regulating the accumulation of mycotoxins. Maor et al. [18] found that ambient pH regulated ochratoxin A biosynthesis in *Aspergillus carbonarius*. Similarly, Jimdjio et al. [9] found that ambient pH influenced the accumulation of patulin in *P. expansum* by influencing patulin’s biosynthesis. Therefore, for pathogens, the appropriate ambient pH value can activate the activity of CWDEs and promote the accumulation of mycotoxins, enhancing their capability to infect host plants.

Our previous study indicated that ambient pH significantly affected the pathogenicity of *F. sulphureum* during infection of muskmelon fruit. However, how pH affects the pathogenicity of *F. sulphureum* by regulating the activity of CWDEs and whether pH has a regulatory effect on the biosynthesis of mycotoxins in *F. sulphureum*-inoculated muskmelon fruit have not been reported. Therefore, the purpose of this study was: (1) to clarify the effect of ambient pH on pathogenicity in *F. sulphureum*-infected muskmelon; (2) to investigate the effects of different ambient pH on spore germination and germ tube growth of *F. sulphureum*; and (3) to determine the extent of spore suspension with different pH values on the CWDE activity of muskmelon fruit. Finally, the impact of ambient pH on DAS accumulation and the relative expression of genes involved in the DAS biosynthetic pathway were analyzed.

## 2. Materials and Methods

### 2.1. Fungal Strain and Muskmelon Fruit

*Fusarium sulphureum* was provided by Gansu Agricultural University, Lanzhou, China.

Muskmelon fruit (cv. “honeydew melon”) of commercial maturity was collected from the plantation base of Qingbaishi Town, located in the north of the Yellow River in Lanzhou, Gansu Province, in July 2021. Muskmelon fruit of good quality and without mechanical damage was selected and transported to the laboratory on the same day and stored at 5–8 °C, 85–90% RH for use.

### 2.2. Preparation of Spore Suspensions with Different pH and Fruit Inoculation

The *F. sulphureum* strain was cultured for 7 days in a 25 °C incubator. Sterile distilled water was buffered with 0.2 M Na_2_HPO_4_·12H_2_O and 0.1 M C_6_H_8_O·7H_2_O, and the pH was adjusted to 3, 5, 6, 7, and 9 using pH meter to prepare a spore suspension with a concentration of 1 × 10^6^ spores/mL. According to our previous research results (the most favorable pH for *F. sulphureum* was 6), a pH value of 6 was used as a control during the whole experiment.

The method of inoculating muskmelon with *F. sulphureum* followed Jimdjio et al. [9] with slight modification. The muskmelon fruit was immersed in 1% NaClO_3_ solution for 5 min for surface disinfection after washing with water, then were allowed to air-dry. Subsequently, the fruit was surface-disinfected with 75% alcohol. Then, four inoculated holes were drilled at the equator of the fruit to a depth of 3 mm with a diameter of 3 mm with a sterilizing iron nail, and 20 μL spore suspensions with different pH were inserted into the inoculated holes. The inoculated fruit was kept at a temperature of 14–18 °C and humidity of 85–90%. From the beginning of inoculation, suspensions buffered with 0.2 M Na_2_HPO_4_·12H_2_O and 0.1 M C_6_H_8_O·7H_2_O (pH 3, 5, 6, 7, and 9) were injected into the inoculation holes of the fruit every 12 h to maintain its pH until the end of our sampling. A total of 20 fruit were employed for each treatment, and each group of experiments was repeated at least three times, so a total of 300 fruit were required.

### 2.3. Assay of the Lesion Diameter and DAS Accumulation

The diameter of lesions (r) was measured at 0, 1, 3, 5, and 7 days post inoculation (DPI), and the lesion area was calculated based on πr^2^. Samples (4.0 g) from the junction of diseased and healthy tissue were collected, quickly frozen in liquid nitrogen, ground into powder, and stored for further use. The decaying tissue at the lesion was cut with a sterile steel knife at 7 DPI and at −80 °C for the determination of DAS content. The determination of DAS content was based on our previous publication [2]. One gram of frozen muskmelon fruit tissue was taken, then extracted with ethyl acetate (1:2, *v*/*v*) twice. The organic phase was combined to remove the ethyl acetate and subsequently dried with nitrogen stream. The dried residues were redissolved in 2 mL acetonitrile for UPLC-MS/MS analysis.

DAS content was analyzed with UPLC-MS/MS (Waters Acquity Ultra Performance LC system, Waters, Milford, MA, USA). The column of a ZORBAX Eclipse Plus C18 was used, and the column was maintained at 40 °C. The linear gradient elution was performed by starting with 95% of mobile phase A (0.1% formic acid aqueous solution) and 5% of mobile phase B (acetonitrile), then changing to 38% of mobile phase A and 62% of mobile-phase B in 1.8 min, subsequently shifted to 95% of mobile phase B within 0.5 min and maintained, then the initial proportion was recovered for 1 min. The flow rate was 0.4 mL min^−1^, and the injection volume was 5.0 μL. In sum, 20 fruit were employed for each treatment, and each group of experiments was repeated at least three times, so a total of 300 fruit were required.

### 2.4. Effects of Ambient pH on Spore Germination and Germ Tube Length

The pH values of PDA medium were adjusted to 3, 5, 6, 7, and 9 with 0.2 M Na_2_HPO_4_·12H_2_O and 0.1 M C_6_H_8_O·7H_2_O buffer, respectively. Spore suspensions of *F. sulphureum* were prepared (1 × 10^6^ spores/mL), and 2 μL of each suspension was inoculated on PDA medium with different pH values. The spore germination and germ tube length were observed and measured under a microscope (CX21FS1C, Olympus, Beijing, China) at 4, 6, and 8 h, respectively.

### 2.5. Effect of Ambient pH on Mycelial Biomass and Mycelial Morphology of F. sulphureum

*F. sulphureum* spore suspension (2 μL, 1 × 10^6^ spores/mL) was inoculated in PDB medium with different pH values and cultured at 25 °C at a speed of 220 rpm for 4 days. Mycelia were collected and their dry weight measured.

Mycelium morphology was observed according to the method of Han et al. [19]. After 3 days of *F. sulphureum* culture, mycelium growth was observed under a microscope.

### 2.6. Effect of Ambient pH on Relative Gene Expression Related to Spore Germination, Sporulation, and DAS Biosynthesis

To assess relative gene expression related to spore germination and sporulation, after *F. sulphureum* had been cultured for 3 days, mycelia were collected and the relative expression of *FsbrlA*, *FsabaA*, *FsvosA*, and *FswetA* genes associated with spore germination and sporulation was determined. For relative gene expression related to DAS biosynthesis, after the muskmelon had been inoculated for 7 days, fruit tissue containing mycelia at the junction of disease–health was taken for DAS biosynthesis-related gene expression determination. Primer sequences are shown in Supporting Information Tables S1 and S2.

The determination of relative gene expression related to spore germination, sporulation, and DAS biosynthesis was based on our previous publication [20]. After *F. sulphureum* had been cultured on a PDA plate for 5 days, mycelia were collected and the expression of *FsbrlA*, *FsabaA*, *FsvosA* and *FswetA* genes associated with spore germination and sporulation analyzed according to the manufacturer’s instructions. Total RNA from *F. sulphureum* mycelium was extracted using the TRNzol Universal Total RNA Extraction Kit (Tiangen Biotech, Beijing, China). RNA structural integrity was detected by 1% agarose gel electrophoresis, and the concentration and purity were checked using an Ultramicro UV-vis spectrophotometer (Kojima Tsuki Manufacturing Institute, Kyoto, Japan). First-strand cDNA was synthesized according to the instructions provided with a gDNA Eraser reverse-transcription kit (Takara, Kusatsu-shi, Japan, RR047A), which was used for the subsequent real-time quantitative PCR (RT-qPCR). The primer sequences for the RT-qPCR assay are listed in Table S2. RT-qPCR was performed using SYBR^®^ Green Premix Pro Taq HS Premix (Rox plus, AG11718) according to the manufacturer’s instructions. The relative expression levels of genes were calculated by the 2^−ΔΔCt^ method with actin and β-tubulin genes as the reference [21].

### 2.7. Effect of Ambient pH on the Activity of CWDEs in Inoculated Fruit

#### 2.7.1. Extraction of Crude Enzyme Solution

Crude enzyme solutions of PMG, PG, Cx, β-Glu, PME, and PL were extracted according to the method of Wang et al. [22]. The crude enzyme solutions of PGTE and PMTE were extracted according to the method of Reinehr et al. [23].

The extraction of PMG, PG, Cx, and β-Glu was performed based on reference [16]: 1.0 g frozen tissue was taken, ground under liquid nitrogen, then 2 mL 5% ethanol was added to the tissue, transferred to a 3 mL centrifuge tube, centrifuged at 4 °C, 10,000× *g* for 10 min, the supernatant removed, 1 mL 80% ethanol added to the precipitation, centrifuged, 1.5 mL of extraction solution added after 20 min, and then the supernatant was centrifuged to collect the crude enzyme solution.

The extraction of PME, PL, PGTE, and PMTE was performed based on reference [17] PME crude enzyme solution extraction: 1.0 g frozen muskmelon fruit tissue was quickly ground under liquid nitrogen, 5 mL 8.8% NaCl solution was added to the tissue to form a homogenate, then centrifuged at 4 °C, 10,000× *g* for 10 min to obtain the supernatant. Then, the pH of the supernatant was adjusted to 7.5 with sodium hydroxide to obtain the crude PME crude enzyme solution.

PL crude enzyme solution extraction: 1.0 g frozen muskmelon fruit tissue was taken and rapidly ground under liquid nitrogen, then 3 mL of Tris-HCl buffer was added to the tissue, centrifuged at 4 °C, 10,000× *g* for 10 min, and the supernatant collected.

PGTE and PMTE crude enzyme solution extraction: 1.0 g of frozen muskmelon fruit tissue was taken, then 4 mL of extraction buffer (Tris-HCl) added to the tissue and centrifuged at 4 °C, 10,000× *g*, for 10 min. The supernatant was collected to obtain the crude enzyme solutions of PGTE and PMTE.

#### 2.7.2. Enzyme Activity Assay

Determination of PME, PG, PMG, PL, Cx, and β-Glu activities were based on the method of Wang et al. [22]. Determination of PME activity: 0.5 mL of the crude enzyme solution extracted was taken, 2 mL of pectin solution and 0.3 mL of bromothymol orchid added, the reaction of the system maintained for 2 min, then the absorption value of the mixture solution was determined.

Determination of PG activity: 0.5 mL of the crude enzyme solution extracted was taken, then 0.5 mL of 10 g/L polygalacturonic acid solution and 1.0 mL of acetate–sodium acetate buffer (pH 5.5) were added to the crude enzyme solution and the reaction maintained in a water bath at 37 °C for 1 h. After 1 h of reaction, 1.5 mL of 3,5-dinitrosalicylic acid solution was added to the reaction solution, the reaction maintained in a boiling water bath for 5 min, then the reaction solution was cooled immediately. Finally, 2 mL of distilled water was added to determine the absorption value of the mixture at 540 nm.

Determination of PMG activity: 0.5 mL of the extracted crude enzyme solution was taken, 0.5 mL of 10 g/L pectin solution and 1.0 mL of acetoacetate sodium buffer (pH 5.5) added, then the whole reaction system was kept in a water bath of 37 °C for 1 h. Then, 1.5 mL of 3,5-dinitrosalicylic acid solution was added to the reaction solution and immediately placed into a boiling water bath. The water bath was cooled after 5 min. Finally, 2 mL of distilled water was added to determine the absorbance value of the mixture at 540 nm.

Determination of PL activity: 2.0 mL 0.5% pectin solution was taken, heated in a water bath at 40 °C for 5 min, 0.5 mL crude pectin lyase added to the pectin solution, water bathed for 10 min again, then 0.5 mL of the mixture was taken and added to 4.5 mL 0.01 mol/L HCl solution. After the reaction system was completely mixed, the absorbance was measured at 235 nm.

Determination of Cx activity: 1.0 mL of the extracted crude enzyme solution was taken, 1.5 mL of 10 g/L sodium carboxymethyl cellulose (CMC) solution added, heated in a water bath at 37 °C for 1 h, 1.5 mL of 3,5-dinitrosalicylic acid quickly added to the crude enzyme solution, boiling water bath for 5 min, and 2 mL of distilled water was added after cooling. Finally, the absorbance was measured at 540 nm.

Determination of β-Glu activity: 0.5 mL of the extracted crude enzyme solution was taken, then 1.5 mL of 10 g/L salicylate solution was added and maintained for reaction in a water bath at 37 °C for 1 h. Subsequently 1.5 mL of 3,5-dinitrosalicylic acid solution was quickly added to the crude enzyme solution, the reaction maintained in a boiling water bath for 5 min, and 2 mL of distilled water was added after cooling. Finally, the absorbance was measured at 540 nm. The activity of PME, PG, PMG, PL, Cx, and β-Glu are expressed as U/g FW.

The determination of PGTE and PMTE activities was based on the method of Reinehr et al. [23]: 1.0 mL of the extracted crude enzyme solution was taken, then 1.0 mL of 50 mmol/L Gly NaOH buffer (pH 9.0), 1.0 mL of substrate, and 1.0 mL of 3 mmol/L CaCl_2_ solution were added to the crude enzyme solution. Once the reaction system was completely mixed, it was kept in a water bath at 30 °C for 5 min, and the absorbance before and after the reaction was measured at 232 nm. PGTE and PMTE activities are expressed as U/g FW.

### 2.8. Statistical Analysis

Experiments were carried out at least 3 times. The mean value and standard deviation of all data were calculated by Excel 2010, and the significance of differences was analyzed by SPSS 21.0 (ANOVA) (*p* < 0.05). Origin 9.0 was employed to make figures.

## 3. Results

### 3.1. Influence of Ambient pH on the Pathogenicity of F. sulphureum and DAS Accumulation in Muskmelon

**Figure 1:**
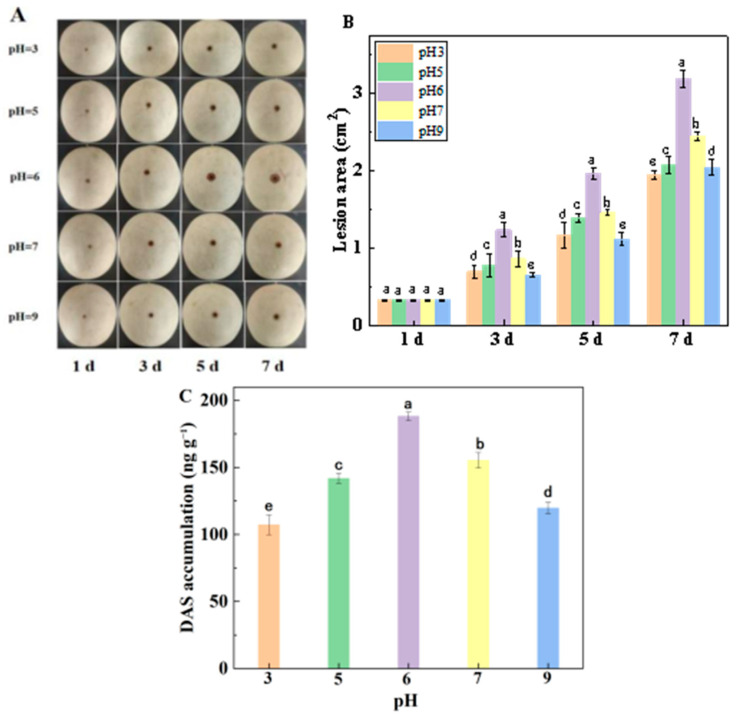
Effects of ambient pH on the disease development and DAS accumulation in muskmelon fruit inoculated with *F. sulphureum*. Picture of the fusarium rot of muskmelon (**A**), lesion area of the fusarium rot of muskmelon (**B**), and DAS accumulation (**C**). Bars represent standard error. Different letters indicate significant difference (*p* < 0.05).

With the prolongation of culture time after inoculation, the lesion areas of muskmelon infected with spore suspensions with different pH values showed an increasing trend, and ambient pH significantly impacted the disease expansion of the muskmelon infected with *F. sulphureum*. For instance, the lesion area of the muskmelons infected with spore suspensions with pH 7, 5, 9, and 3 were 2.4, 2.1, 2.0, and 1.9 cm^2^, respectively, at 7 DPI. Compared with pH 6 inoculation (3.2 cm^2^), the lesion area decreased by 23.55%, 35.18%, 35.99%, and 39.14%, respectively (Figure 1A,B). Accordingly, ambient pH also significantly influenced DAS accumulation in muskmelon infected with *F. sulphureum*, with a DAS content of 188.2 ng/g at pH 6. Compared with pH 6, the DAS content of pH 7, 5, 9, and 3 decreased by 17.43%, 24.60%, 36.40%, and 43.04%, respectively (Figure 1C).

### 3.2. Effects of Ambient pH on Spore Development and Gene Expression Associated with Spore Development of F. sulphureum

**Figure 2:**
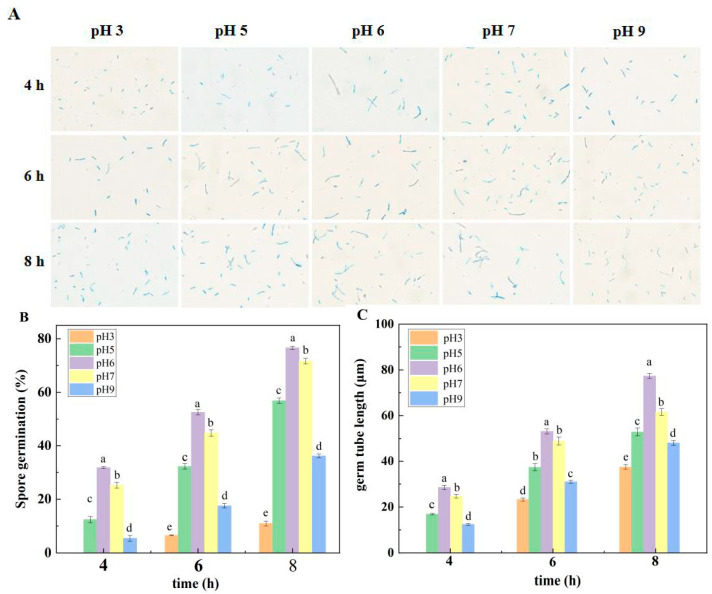
Effect of ambient pH on spore germination (**A**,**B**) and germ tube length (**C**). Bars represent standard error. Different letters indicate significant difference (*p* < 0.05).

Different ambient pH values had a remarkable effect on spore germination and germ tube length. As can be seen in Figure 2A,B, the spore germination rate and sporulation of *F. sulphureum* at pH 6 were the highest, followed by pH 7, then pH 5, 9, and 3. For instance, the spore germination rate at pH 6 was 6.89%, 34.78%, 112.11%, and 602.28% higher than pH 7, 5, 9, and 3, respectively. A similar trend was observed for *F. sulphureum* germ tube growth, and the fastest growth rate was found at pH 6, with the growth rate of germ tubes inhibited to varying degrees at other pH values. For instance, the inhibition rates were 25.51%, 46.30%, 60.82% and 106.10%, respectively, compared with pH 6 (Figure 2C).

**Figure 3:**
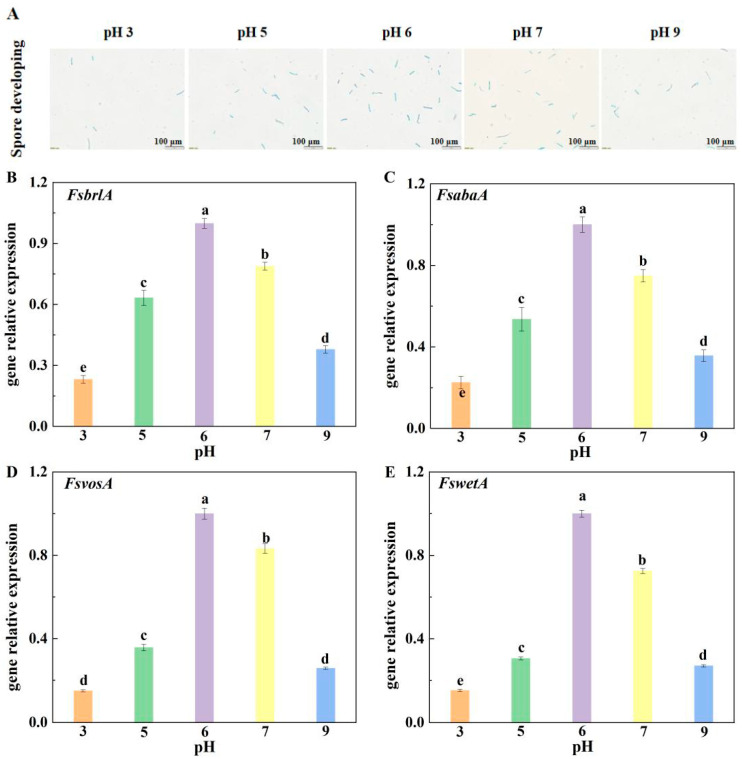
Effect of ambient pH on spore development (**A**) and relative expression of *FsbrlA* (**B**), *FsabaA* (**C**), *FsvosA* (**D**), and *FswetA* (**E**). Bars represent standard error. Different letters indicate significant difference (*p* < 0.05).

As shown in Figure 3A, there were significant differences in the sporulation of *F. sulphureum* after 5 days of growth on different pH media. At pH 6, the sporulation of *F. sulphureum* was the highest, followed by pH 7, 5, 9, and 3. *FsBrlA*, *FsabaA*, *FswetA*, and *FsvosA* were the key genes involved in spore germination and sporulation of *F. sulphureum*. The gene expression results showed that ambient pH markedly affected the relative expression of *FsbrlA*, *FsabaA*, *FsvosA*, and *FswetA*. Among the different pH treatments, the spore development and expression of *FsbrlA*, *FsabaA*, *FsvosA*, and *FswetA* were the highest, at pH 6 (Figure 3B–E). Compared with pH 6, the relative expression of *FsbrlA*, *FsabaA*, *FsvosA* and *FswetA* were significantly down-regulated at other pH values, indicating that the relative expression levels of *FsbrlA*, *FsabaA*, *FsvosA* and *FswetA* were significantly down-regulated at other pH values when compared with that at pH 6, thus reducing the spore germination rate and sporulation of *F. sulphureum.*

### 3.3. Effects of Ambient pH on Mycelial Dry Weight and Mycelial Morphology

**Figure 4:**
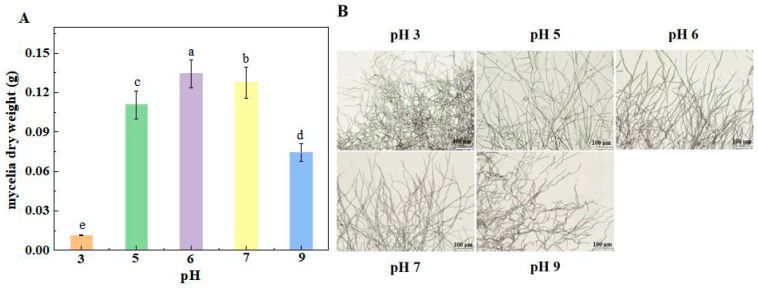
The effect of ambient pH on mycelium dry weight (**A**) and the colony edges on *F. sulphureum* under light microscopy (**B**). Bars represent standard error. Different letters indicate significant difference (*p* < 0.05).

Inoculation of spore suspensions with different pH had significant effects on the dry weight of mycelia. The dry weight of mycelia infected with spore suspension with pH 6 was the largest, followed by pH 7, 5, and 9, and the dry weight of mycelia infected with spore suspension with pH 3 was the lowest. For instance, the dry weight of mycelia inoculated at pH 6 was 10.29%, 18.16%, 80.16%, and 107.17% higher than those infected at other pH values (Figure 4A).

The results showed that the morphology of the mycelia at the edge of the colony was significantly different after inoculating spore suspensions with different pH values. The colony-edge hyphae treated with pH 6 were dense and neat, and the surface was smooth and full. However, the hyphae at the edge of the colonies treated with pH 3 and pH 9 became curved and messy. The mycelia became sparser and slenderer after inoculation with pH 5 and pH 7 spore suspensions (Figure 4B).

### 3.4. Effects of Ambient pH on CWDE Activities

#### 3.4.1. Effects of Ambient pH on PME, PG, PMG, and PL Activities

**Figure 5:**
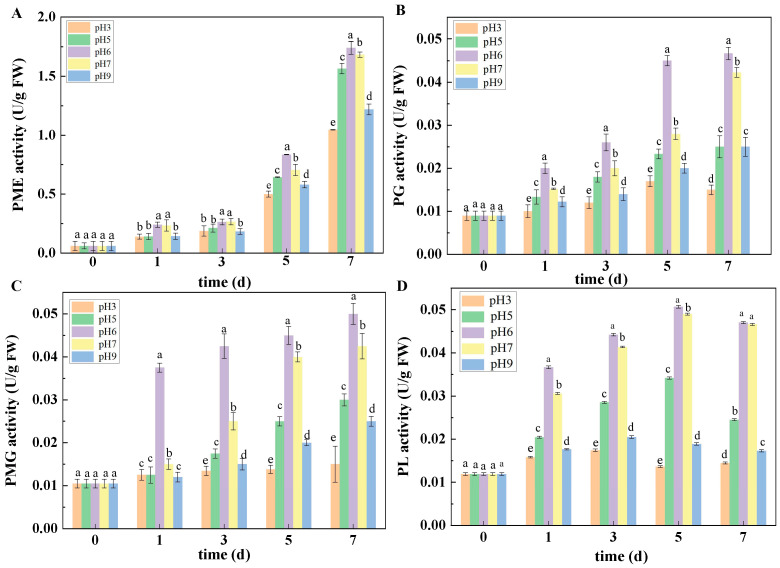
The effect of ambient pH on the activities of PME (**A**), PG (**B**), PMG (**C**), and PL (**D**). Bars represent standard error. Different letters indicate significant difference (*p* < 0.05).

As shown in Figure 5, the inoculation of spore suspensions with different pH values had significant effects on PME, PG, PMG, and PL activities in inoculated fruit. In general, the activities of PME, PG, PMG, and PL in muskmelon infected with spore suspensions at pH 6, 5, 7, and 9 increased with the increase in culture time after inoculation; however, the change trend of PME, PG, PMG, PL activities was not obvious when infected with spore suspensions at pH 3. At 7 DPI, the PME, PG, PMG, and PL activities of the muskmelon infected with pH 6 spore suspension were the highest, followed by those inoculated with pH 7, 5, and 9 spore suspensions, and the lowest PME, PG, PMG, and PL activities in muskmelon infected with spore suspensions at pH 3 were observed (Figure 5A–D).

#### 3.4.2. Effects of Ambient pH on PGTE, PMTE, Cx, and β-Glu Activities

**Figure 6:**
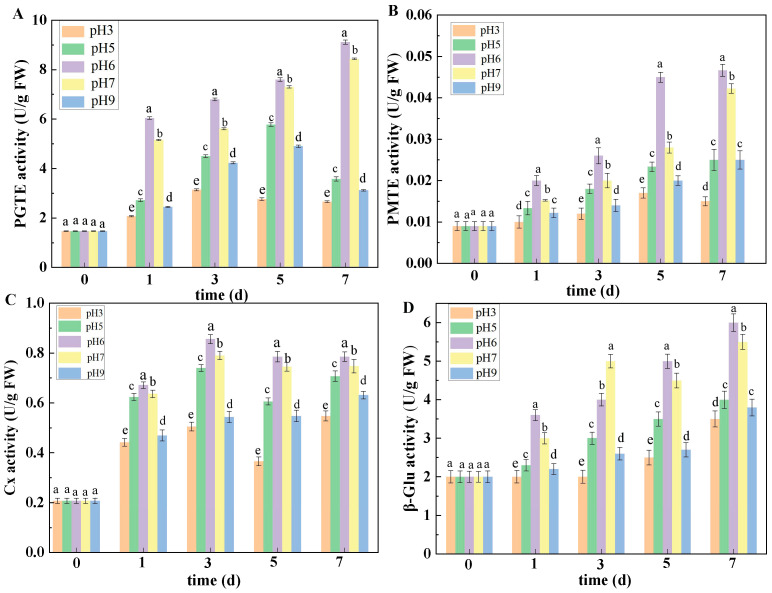
The effect of ambient pH on the activities of PGTE (**A**), PMTE (**B**), Cx (**C**), and β-Glu (**D**). Bars represent standard error. Different letters indicate significant difference (*p* < 0.05).

The inoculation of spore suspensions at different pH levels had significant effects on PGTE and PMTE activities in fruit. With the prolongation of inoculation time, PGTE and PMTE activities in fruit showed an overall increasing trend. Among different pH treatments, the PGTE and PMTE activities in fruit inoculated with spore suspensions at pH 6 were the highest, followed by those at pH 7, 5, and 9, and the lowest PGTE and PMTE activities in muskmelon infected with spore suspensions were observed at pH 3. At 7 DPI, the PGTE and PMTE activities in fruit treated with pH 6 reached the maximum value, and were higher than those in muskmelon infected with spore suspensions with other pH values (Figure 6A,B).

Similarly, inoculation with spore suspensions of different pH values also had a significant effect on Cx and β-Glu activities in fruit, and Cx and β-Glu activity showed an increasing trend with time. Among the different pH values, the Cx activity in fruit inoculated with spore suspension with pH 6 was the highest, followed by pH 7, 5, and 9, and the Cx activity at pH 3 was the lowest. At 7 DPI, the Cx activity of pH 6 inoculation reached the maximum value, and was significantly higher than those of other pH value inoculations, with increases of 5.89%, 43.26%, 89.32%, and 120.34%, respectively (Figure 6C). β-Glu activity at pH 6 treatment also showed the maximum value, and was significantly higher than those at other pH values after inoculation, with increases of 19.67%, 34.00%, 56.72%, and 61.33%, respectively, at 7 DPI. The activity of β-Glu was the lowest in muskmelon tissue infected with pH 3 spore suspension (Figure 6D).

### 3.5. Effects of Ambient pH on the Relative Expression of Genes Involved in DAS Biosynthesis

**Figure 7:**
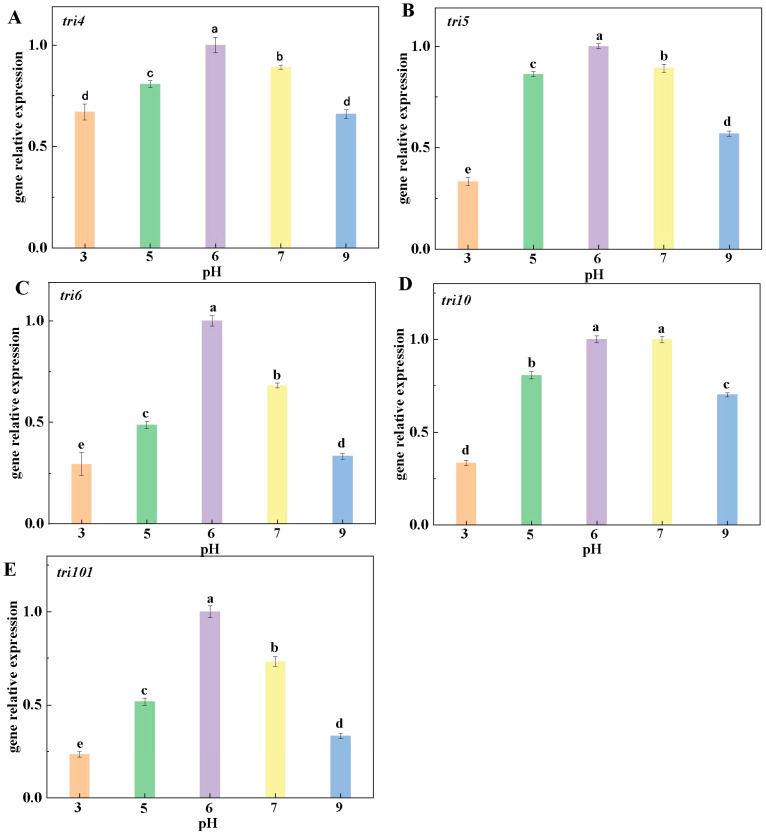
The effect of ambient pH on the relative expression of *tri4* (**A**), *tri5* (**B**), *tri6* (**C**), *tri10* (**D**), and *tri101* (**E**) involved in DAS biosynthesis pathway on muskmelon. Bars represent standard error. Different letters indicate significant difference (*p* < 0.05).

The results of genes expression related to DAS biosynthesis echoed DAS accumulation. At pH 6, the expression of *tri4*, *tri5*, *tri6*, *tri10* and *tri101* was significantly higher than those at other pH values (Figure 7A–E). These results suggested that the inoculations with other pH-value spore suspensions in fruit down-regulated the DAS biosynthetic pathway, thereby reducing DAS accumulation in the muskmelon infected with *F. sulphureum*.

## 4. Discussion

In this study, it was found that the inoculation of spore suspensions with different pH values had a significant effect on disease expansion and DAS accumulation in muskmelon fruit. Among these, the lesion area in the muskmelon infected with spore suspension with pH 6 was the greatest, and the smallest lesion area of the inoculated fruit was observed at pH 3, indicating that the ambient pH 6 was more conducive to pathogenicity for *F. sulphureum*. This result is similar to the report by Wang et al. [24], who suggested that ambient pH 7 was beneficial to pathogenicity for *Trichothecium roseum* to fruit. In addition, Shi et al. [25] also observed that when pH was 6, *F. sporotrichioides* grew faster in PDB medium, and too acidic or too alkaline an ambient pH was not conducive to growth or pathogenicity of *F. sporotrichioides*. Furthermore, the highest DAS accumulation was also observed in the muskmelon infected with spore suspension with pH 6, and the lowest DAS content in the inoculated fruit was observed at pH 3. This result was consistent with the report by Jimdjio et al. [9], who suggested that inoculation with spore suspension with pH 5 not only resulted in the most serious disease but also an accumulation of the highest level of patulin in fruit. The possible mechanism of action was attributed to two factors from in vitro and in vivo experiments.

An important environmental factor, ambient pH significantly affects the growth and development of pathogenic fungi in vitro [26]. In this study, mycelial dry weight, spore germination rate, and germ tube length of *F. sulphureum* at pH 7, 5, 9, and 3 were significantly inhibited compared with those at pH 6, and the growth rate of *F. sulphureum* was the slowest at pH 3 and 9, which was similar to the results of Wang et al. [24], who found that the colony diameter and spore germination rate of *T. roseum* were significantly inhibited under a strong acid or alkali environment. The mycelial growth and conidial formation of pathogenic fungi under different pH were closely related to the morphology of pathogenic fungi. Our study showed that the mycelium morphology of the colony edge was significantly affected by ambient pH. The mycelium inoculated with a spore suspension at pH 6 had clear structure and smooth and full surfaces. The mycelium inoculated with pH 3 was thickened compared with that inoculated with pH 6. The mycelium inoculated with pH 5 became obviously thinner. pH 9 inoculation significantly disrupted the marginal structure of the mycelium, and the mycelium became dense and even intertwined with other branches. This phenomenon was consistent with the observation by Jimdjio et al. [9], who also found that mycelium morphology of colony edges was more obvious and branches were longer and sparser at pH 5.0 and 7.0, while the edges were shorter and less dense at pH 2.5 and 8.5. Similarly, Li et al. [26] found that ambient pH affected the intracellular pH and ATP levels of *P. expansum* spores, and the germination of *P. expansum* spores was significantly inhibited at pH 2.0 and 8.0. The reason could be that strong acids or bases destroy chromosomes and proteins’ DNA, thus inhibiting conidium growth and changing colony morphology.

In this study, compared with other treatment groups, the relative expression levels of *FsbrlA*, *FsabaA*, *FswetA*, and *FsvosA* in the mycelium inoculated with pH 6 spore suspension were up-regulated and displayed the highest expression, which made *F. sulphureum* produce the most spores and have the strongest growth and development ability at pH 6, followed by those infected with spore suspensions at pH 7, 5, and 9. Expression levels at pH 3 were the lowest, and accordingly, *F. sulphureum* had the lowest sporulation and the weakest growth and development ability at pH 3. Therefore, we speculate that ambient pH regulates the sporulation of pathogens by affecting the expression of sporulation-related genes, thereby reducing the susceptibility to pathogenicity of muskmelon fruit.

The secretion and expression of pathogenic factors was also influenced by ambient pH to a certain extent. As we know, when pathogens infect host plants, fungi will secrete plenty of CWDEs to degrade cell walls. CWDEs can degrade pectin substances of host plant cell walls, thus breaking plant defenses and promoting infections with pathogenic fungi [27]. In the present study, the results showed that the activities of PMG, PL, PME, PG, Cx, β-Glu, PGTE, and PMTE in muskmelon inoculated with the pH 6 spore suspension were higher than those in muskmelon infected with the other pH-value spore suspensions. The above results indicated that ambient pH can regulate the secretion of CWDEs of pathogenic fungi, which is similar to the results of Juntachai et al. [28], who indicated that ambient pH plays a promoting role in the secretion of *Malassezia furfur* lipase. It has been reported that the CWDEs produced by *Colletotrichum gloesporioides* and *C. coccodes* during fruit infection were related to their pathogenicity [17,29,30,31]. The CWDEs secreted by *P. expansum*, *P. digitatum*, and *P. italicum* were more conducive to the infection of apple and citrus fruit with these pathogens [32]. Other studies have also found that *PG* genes play an important role in the infection of different fruit by *Botrytis cinerea* and *Alternaria citri* [33,34]. In brief, ambient pH can regulate the secretion of CWDEs of pathogenic fungi during the infection process, which play an important role in the pathogenesis of fungi.

The occurrence of fusarium rot caused by *F. sulphureum* is accompanied by DAS accumulation. DAS is one kind of trichothecene, the biosynthesis of which begins with farnesyl pyrophosphate (FPP). Under the action of the key genes *tri4* and *tri5* and main regulatory genes *tri6*, *tri10*, and *tri101*, FPP is synthesized through a series of complex oxygenation, isomerization, cyclization, and esterification reactions under a series of enzymatic reactions. The results of this study showed that compared with the ambient pH 6 treatment, expression of *tri* genes in the DAS biosynthesis pathway was down-regulated under other pH conditions, thereby reducing the accumulation of DAS in fruit inoculated with *F. sulphureum*. This result was similar to Jimdjio’s report [9], whose results showed that the expression of patulin biosynthesis-related genes could be down-regulated by ambient pH. Accordingly, the biosynthesis pathway of patulin was inhibited, thereby reducing the patulin accumulation in fruit infected with *P. expansum*. In brief, the above results show that ambient pH affected the biosynthesis of DAS by down-regulating the expression of genes related to the biosynthesis pathway of DAS, thereby reducing DAS accumulation in inoculated fruit.

## 5. Conclusions

**Figure 8:**
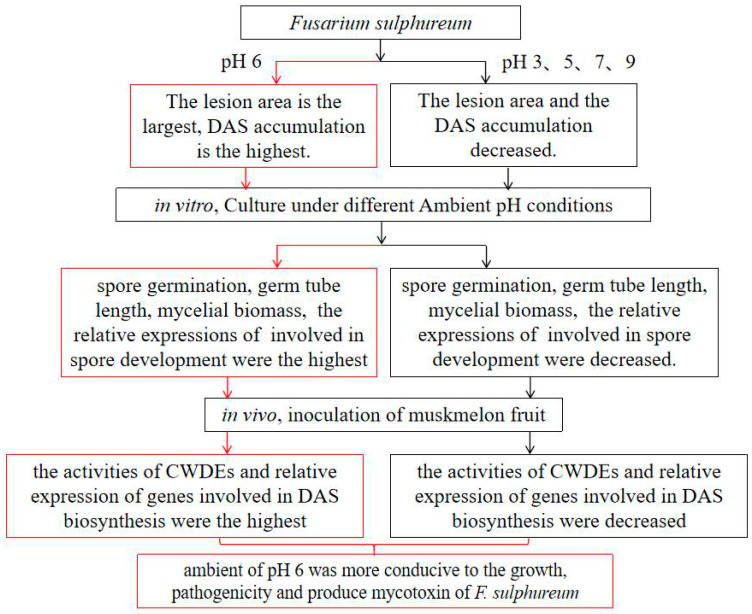
Ambient pH 6-modulated pathogenicity and DAS accumulation in muskmelon fruit infected with *F. sulphureum* and possible mechanism of action (compared to other pH values). Red indicates pH 6, and black indicates other pH values.

The results showed that different ambient pH had significant effects on the growth, development, and pathogenicity of *F. sulphureum*. A possible mechanism of the effect of ambient pH on the pathogenicity and DAS toxin accumulation of *F. sulphureum* is shown in Figure 8. Compared with other pH values, with ambient pH 6, the pathogenicity of *F. sulphureum* was the strongest, the lesion area of muskmelon was the largest, and the DAS content was the highest. The results of in vitro experiments showed that the growth and development of *F. sulphureum* was the fastest at pH 6. In vivo, the muskmelon inoculated with the pH 6 spore suspension had the highest CWDEs activity and DAS synthesis-related gene expression. These results suggest that ambient pH regulates the pathogenicity of *F. sulphureum* in muskmelon fruit by influencing its growth and development, secretion of CWDEs, and biosynthesis of DAS. These findings will help in understanding the effects of ambient pH on the growth of *F. sulphureum* and the accumulation of DAS toxins, and support the development of successful methods to control the infection of muskmelon fruit by *F. sulphureum*.

## Data Availability

No data was used for the research described in the article.

## Supplementary Materials

The following supporting information can be downloaded at https://www.mdpi.com/article/10.3390/jof10110765/s1, Table S1: The primers used for Real-time PCR of genes relative expression related to spore germination and sporulation; Table S2: The primers used for Real-time PCR of genes DAS biosynthesis related gene expression.

## References

[1] Li L., Liu Q., Xue H., Bi Y., Raza H., Zhang R., Carelle J.K., Peng H., Long H., Prusky D. (2022). Acetylsalicylic acid (ASA) suppressed Fusarium rot development and neosolaniol (NEO) accumulation by activating phenylpropane metabolism in muskmelon fruit. Eur. J. Plant Pathol..

[2] Li L., Xue H., Bi Y., Zhang R., Kouasseu C.J., Liu Q., Nan M., Pu L., Prusky D. (2021). Ozone treatment inhibits dry rot development and diacetoxyscirpenol accumulation in inoculated potato tuber by influencing growth of *Fusarium sulphureum* and ergosterol biosynthesis. Postharvest Biol. Technol..

[3] Jin G., Wu X., Cui G., Liu L., Kuang H., Xu C. (2020). Development of an ic-ELISA and Immunochromatographic Strip Assay for the Detection of Diacetoxyscirpenol in Rice. ACS Omega.

[4] Selvig K., Alspaugh J.A. (2011). pH response pathways in fungi: Adapting to host-derived and environmental signals. Mycobiology.

[5] Mah J.-H., Yu J.-H. (2006). Upstream and downstream regulation of asexual development in *Aspergillus fumigatus*. Eukaryot. Cell.

[6] Tao L., Yu J.-H. (2011). AbaA and WetA govern distinct stages of *Aspergillus fumigatus* development. Microbiology.

[7] Ni M., Yu J.H. (2007). A novel regulator couples sporogenesis and trehalose biogenesis in *Aspergillus nidulans*. PLoS ONE.

[8] Zhang X., Hou X., Xu D., Xue M., Zhang J., Wang J., Yang Y., Lai D., Zhou L. (2023). Effects of carbon, nitrogen, ambient pH and light on mycelial growth, sporulation, sorbicillinoid biosynthesis and related gene expression in *Ustilaginoidea virens*. J. Fungi.

[9] Jimdjio C.K., Xue H., Bi Y., Nan M., Li L., Zhang R., Liu Q., Pu L. (2021). Effect of ambient pH on growth, pathogenicity, and patulin production of *Penicillium expansum*. Toxins.

[10] Kars I., Kan J. (2007). Extracellular enzymes and metabolites involved in pathogenesis of *Botrytis*. Botrytis: Biology, Pathology and Control.

[11] Jia Y.J., Feng B.Z., Sun W.X., Zhang X.G. (2009). Polygalacturonase, pectate lyase and pectin methylesterase activity in pathogenic strains of *Phytophthora capsici* Incubated under different conditions. J. Phytopathol..

[12] Eshel D., Miyara I., Ailing T., Dinoor A., Prusky D. (2002). pH Regulates endoglucanase expression and virulence of *Alternaria alternata* in persimmon fruit. Mol. Plant-Microbe Interact..

[13] Li G., Dong B., Li Y., He Y., Luo Q. (2023). pH regulates the pathogenicity and extracellular enzyme activity of *Gilbertella persicaria* on pitaya fruit. Eur. J. Plant Pathol..

[14] Shi W., Han Z., Wu A.B., Wang Z.P. (2015). Effect of temperature and pH on the growth and mycotoxins production of various *Fusarium species*. Sci. Technol. Food Ind..

[15] Marín-Rodríguez M.C., Orchard J., Seymour G.B. (2002). Pectate lyases, cell wall degradation and fruit softening. J. Exp. Bot..

[16] De Cal A., Sandín-España P., Martinez F., Egüen B., Chien-Ming C., Lee M., Melgarejo P., Prusky D. (2013). Role of gluconic acid and pH modulation in virulence of *Monilinia fructicola* on peach fruit. Postharvest Biol. Technol..

[17] Yakoby N., Kobiler I., Dinoor A., Prusky D. (2000). pH regulation of pectate lyase secretion modulates the attack of *Colletotrichum gloeosporioideson* avocado fruits. Appl. Environ. Microbiol..

[18] Maor U., Sadhasivam S., Zakin V., Prusky D., Sionov E. (2017). The effect of ambient pH modulation on ochratoxin A accumulation by *Aspergillus carbonarius*. World Mycotoxin J..

[19] Han Z., Wang Z., Bi Y., Zong Y., Gong D., Wang B., Li B., Sionov E., Prusky D. (2021). The effect of environmental pH during *Trichothecium roseum* (Pers.:Fr.) link inoculation of apple fruits on the host differential reactive oxygen species metabolism. Antioxidants.

[20] Liu Q., Zhang Q., Xue H., Bi Y., Yang X., Zong Y., Liu Z., Chen J., Prusky D. (2023). *TrPLD1* and *TrPLD2* modulate reactive oxygen species production and pathogenicity in *Trichothecium roseum* infected apple fruit. Postharvest Biol. Technol..

[21] Livak K.J., Schmittgen T.D. (2001). Analysis of relative gene expression data using real-time quantitative PCR and the 2^−ΔΔCT^ method. Methods.

[22] Wang Z.Y., Hu H.M., Gong D., Zhang G.J., Prusky D., Yang B.Y. (2019). Acid-Base property of *Trichothecium roseum* and effect of pH on its extracellular enzyme activities and pathogenicity. Food Sci..

[23] Reinehr R., Görg B., Becker S., Qvartskhava N., Bidmon H.J., Selbach O., Haas H.L., Schliess F., Häussinger D. (2010). Hypoosmotic swelling and ammonia increase oxidative stress by39 NADPH oxidase in cultured astrocytes and vital brain slices. Glia.

[24] Wang B., Han Z., Gong D., Xu X., Li Y., Sionov E., Prusky D., Bi Y., Zong Y. (2022). The pH signalling transcription factor PacC modulate growth, development, stress response and pathogenicity of *Trichothecium roseum*. Environ. Microbiol..

[25] Kramer-Haimovich H., Servi E., Katan T., Rollins J., Okon Y., Prusky D. (2006). Effect of ammonia production by *Colletotrichum gloeosporioides* on *pelB* activation, pectate lyase secretion, and fruit pathogenicity. Appl. Environ. Microbiol..

[26] Li B., Lai T., Qin G., Tian S. (2009). Ambient pH stress inhibits spore germination of *Penicillium expansum* by impairing protein synthesis and folding: A proteomic-based study. J. Proteome Res..

[27] Akimitsu K., Isshiki A., Ohtani K., Yamamoto H., Eshel D., Prusky D. (2004). Sugars and pH: A clue to the regulation of fungal cell wall-degrading enzymes in plants. Physiol. Mol. Plant Pathol..

[28] Juntachai W., Chaichompoo A., Chanarat S. (2020). Ambient pH regulates secretion of lipases in malassezia furfur. Microbiology.

[29] Yakoby N., Beno-moualem D., Keen N.T., Dinoor A., Pines O., Prusky D. (2001). *Colletotrichum gloeosporioides* pelB is an important virulence factor in avocado fruit-fungus interaction. Mol. Mol. Plant-Microbe Interact..

[30] Drori N., Kramer-Haimovich H., Rollins J., Dinoor A., Okon Y., Pines O., Prusky D. (2003). External pH and nitrogen source affect secretion of pectate lyase by *Colletotrichum gloeosporioides*. Appl. Environ. Microbiol..

[31] Ben-Daniel B.H., Bar-Zvi D., Tsror L. (2012). Pectate lyase affects pathogenicity in natural isolates of *Colletotrichum coccodes* and in *pelA* gene-disrupted and gene-overexpressing mutant lines. Mol. Plant Pathol..

[32] Prusky D., McEvoy J.L., Saftner R., Conway W.S., Jones R. (2004). Relationship between host acidification and virulence of *Penicillium* spp. on apple and citrus fruit. Phytopathology.

[33] Ten Have A., Mulder W., Visser J., van Kan J.A. (1998). The endopolygalacturonase gene Bcpg1 is required for full virulence of *Botrytis cinerea*. Mol. Plant-Microbe Interact..

[34] Isshiki A., Akimitsu K., Yamamoto M., Yamamoto H. (2001). Endopolygalacturonase is essential for citrus black rot caused by *Alternaria citri* but not brown spot caused by *Alternaria alternata*. Mol. Plant-Microbe Interact..

