# Supplementary material for: Effects of Ambient pH on the Growth and Development, Pathogenicity, and Diacetoxyscirpenol Accumulation of Muskmelon Fruit Caused by Fusarium sulphureum
Source: J Fungi (Basel). 2024 Nov 3;10(11):765. doi: 10.3390/jof10110765 (PMC11595694; doi:10.3390/jof10110765)
Supplement: Supplementary file 1 [file jof-10-00765-s001.zip › jof-3247764-supplementary.pdf]

Table S1 The primers used for Real-time PCR of genes relative expression related to spore germination and sporulation

Gene	Primer sequences (5'-3')
FsbrlA	F: GGCCATGTGCAAGTGTGACT
	R: ATGTTTGCTTATGGCGCTTGA
FsabaA	F: GAGGATGCCTTTGTCGATTCC
	R: TTCGACCGTGGAGCTTTCC
FswetA	F: ACGGAAGCTGAGCGAAGCT
	R: GGAAACCCTGTTCCAGCAATT
FsvosA	F: GGAGTCGAGTTCACCCACATG
	R: GCGAAGCTTGTACCCCTTCTC
Actin	F: GAGCCTGAGCGTTTCACTATT
	R: AGAGAAGGCCTCGTTGATCT

Table S2 The primers used for Real-time PCR of genes DAS biosynthesis related gene expression

Gene	Primer sequences (5'-3')
Tri4	F:TAAACGCCCGCGAAGTTCACA
	R: TGGTGATGGTTCGCTTCGAG
Tri5	F: TGCAAGTTCTTTGAGCAGGC
	R: CTCCACTAGCTCAATTGAACTTAG
Tri6	F: FAGCGCCTTGCCCCTCTTTG
	R: RAGCCTTTGGTGCCGACTTCTTG
Tri10	F: FTCTGAACAGGCGATGGTATGGA
	R: RCTGCGGCGAGTGAGTTTGACA
Tri101	F: TTGGGTTTGGACTGGGTAAG
	R: TTGCGTACTTTGTCCACTCCT
β-tubulin	F: GGTAACCAAATCGGTGCTGCTTTC
	R: GATTGACCGAAAACGAAGTTG
